# Long-term outcomes after removal of rib stabilization hardware in patients with blunt chest trauma

**DOI:** 10.1007/s00068-025-02858-y

**Published:** 2025-04-29

**Authors:** Maria B. Svec, Helga Bachmann, Aljaz Hojski, Eric F. Macharia-Nimietz, Sandrine V. C. Dackam, Didier Lardinois

**Affiliations:** Department of Thoracic Surgery, University Hospital Basel, Spitalstrasse 21, Basel, 4031 Switzerland

**Keywords:** Blunt chest trauma, Hardware removal, Long-term outcome, Health status

## Abstract

**Purpose:**

The study aim was to investigate the long-term outcomes after hardware removal (HR) in patients with blunt chest trauma. We hypothesized that HR might be beneficial in indicated patients to improve patient health.

**Methods:**

We performed a retrospective single-center study between 2017 and 2023. Descriptive statistics were used for the analysis. One study-specific follow-up visit was conducted, 28 months (range 3–72) after HR. The study-specific health survey used, consisted of four functional dimensions (mobility, self-care, usual activities, mental health) and two symptom dimensions (thoracic pain, chest tightness) which were rated on a numerical scale and compared to the status before HR in four categories (much better to worse).

**Results:**

Of the 28 patients, the average age was 59 years (range 29–83), 12 fractures (1–39) were fixed, and 4 implants (1–11) were used. The indications for HR were persistent thoracic pain (36%), discomfort (25%), chest tightness (21%), hardware dislocation (11%) and hardware infection (7%). HR was performed 18 months (5 days-104 months) after surgery for trauma. Patients with chest tightness and infection exhibited the greatest improvement in symptoms (75%), followed by those with hardware dislocation (61%). The improvement rate in the other 2 groups was 58%. All patients who experienced chest tightness, hardware dislocation and infection were completely satisfied and would undergo HR again.

**Conclusion:**

HR is safe and feasible, resulting in significant symptom relief and improvement in health status in approximately two-thirds of patients. In indicated patients, HR might be performed earlier and more liberally if symptoms are disabling.

**Clinical trial registration number:**

NCT06003595 registered on July 18, 2023.

## Introduction

Chest trauma accounts for 25% of all injury-related deaths [1] and is the third most common traumatic cause of death after abdominal and head trauma in polytraumatized patients [2]. Rib fractures occur in up to 39% of patients with blunt chest trauma and 10% of trauma admissions in general [3]. The in-hospital mortality rates reach 7% in patients with multiple rib fractures and 16% for those with flail chest [4]. In accordance with the favorable clinical outcomes of surgical stabilization of rib fractures (SSRF) [5–10], the interest of surgeons in this procedure has increased [7]. At our hospital, we perform chest wall stabilization or rib fixation surgery on approximately 70–80 patients per year using rib clips (approximately 85%), plates with screws (15%) and sometimes a combination of clips and plates. Although SSRF is presumed to be a relatively safe method [11], describing its complications and consequences is important [12–14]. After performing SSRF, the hardware should be left in place unless any complications require unintended hardware removal (HR). To date, the removal of rib stabilization hardware represents an uncommon procedure. It remains unclear whether symptoms such as thoracic pain or discomfort are caused by the trauma itself or by the implanted hardware. Therefore, current management of these symptoms tends to avoid HR [15]. Recent studies have demonstrated that a possible indication for HR is hardware failure [15–21]. Choi et al. [22] published a meta-analysis of hardware failure, which has been defined as a complication causing an unintended HR, prolonged pain, discomfort, or rib fracture nonunion. This condition occurs in 4% of patients, and approximately 60% of these patients require HR [16]. However, the etiology of hardware failure remains only partially explained, and clear indications have not been sufficiently defined. There is currently a lack of studies on systematic long-term outcomes after the removal of rib stabilization hardware.

We hypothesized that HR might be beneficial in indicated patients to improve patient health. The primary objective of our study was to evaluate the patient-reported long-term outcomes of health status, thoracic pain, symptom relief, and patient satisfaction at a study-specific follow-up visit of patients who underwent HR after surgical stabilization of rib fracture. The secondary objective was to identify indications for HR.

## Patients and methods

### Study design

A single-center retrospective study with one study-specific follow-up visit was conducted with 28 patients who underwent HR after SSRF between September 1, 2017, and September 30, 2023.

### Patient screening and recruitment

Patients were identified via hospital records using the International Classification of Diseases (ICD-10-GM Version 2023) [23] with a diagnosis of rib fracture (S22.3-22.5). A total of 41 patients underwent HR during the study period. The inclusion and exclusion criteria have been checked in detail and completed. All patients with blunt chest trauma in our department aged 18 years or older with at least one rib fracture who underwent SSRF and HR without repeated SSRF and who provided written informed consent were included. However, thirteen patients did not fulfill the inclusion criteria, and 28 (4%) were eligible for our study. All eligible patients were invited for one study-specific follow-up visit at least 3 months after HR. They were asked to complete the study-specific health survey including satisfaction with the cosmetic results and the decision for surgery. Patients who could not be reached and those who were contacted but did not want to return for the study-specific follow-up visit were included if a signed general research consent was available, and existing data were retrieved from the medical records and then analyzed.

### Surgical procedure for hardware removal

Our standardized surgical procedure for HR included general anesthesia with perioperative antibiotic prophylaxis without exploratory video-assisted thoracoscopy and optional insertion of a chest tube. There was no need for VATS because we did not expect any indications or benefits of this technique, for example, better localization of implants intended for HR. For HR surgery, we performed a muscle-sparing technique via the previous incision, with or without its excision. Additional techniques were used when lung tissue was interposed, intercostal nerve entrapment was suspected, or neobursa was present.

### Data collection and outcome measures

#### Study health survey part I and part II

At the study-specific follow-up visit, we evaluated patient health status, improvement in health status and satisfaction as patient-reported long-term outcome measures, which was the primary objective of the study. To assess health status, we used a study-specific health survey and additional questions. The study health survey was presented to patients in German or French. The study investigator introduced the instrument to the patients and was present to clarify any uncertainties.

In the first part of the health status survey, questions were asked about the following: 4 functional dimensions at the time of the follow-up visit: mobility, self-care, usual activities, mental health (anxiety/depression); 2 symptom dimensions: thoracic pain at rest and chest tightness; and 3 additional dimensions: resumption of professional activities in previously employed patients, satisfaction with the cosmetic result after HR, and satisfaction with the decision for HR surgery. Global health status consists of the functional dimension and the symptom dimension. In addition, patients were asked about their analgesic use and whether chest tightness was due to trauma or hardware. A numerical rating scale (NRS) ranging from 0 to 10 (in whole numbers) was used because patients were already familiar with this scale from in-hospital pain surveys. A value of 0 was generally defined as “no difficulty present”. A value of 10 was generally defined as “most difficulty present”. A median value was calculated for each question and for each HR indication group. A global health status median value was calculated for each HR indication group.

In the second part of the health status survey, we asked the patient to rate the status of the functional and symptom dimensions at the time of the post-HR follow-up visit compared to the health status before the HR in four categories (worse, no change, slightly better, much better). We determined the percent improvement rate for each question and for each HR indication group as well as the global health status improvement rate for each HR indication group.

#### Retrospective data

The secondary objective of the study was to identify indications for HR. Indications for HR were analyzed in the following predefined groups: persistent thoracic pain, discomfort, chest tightness, hardware dislocation, and hardware infection. We also collected the following retrospective data: patient demographics, trauma-related data (number and location of rib fractures defined by axillary lines), surgery-related data (date of SSRF and HR; number and location of fixed ribs and ribs with hardware removed; hardware type), interval from HR to the study-specific follow-up visit and in-hospital complications related to HR, graded according to the Clavien-Dindo classification [24]. The Clavien-Dindo classification is used for grading complications from grade I to V. The type of therapy required to correct the complication determines the grade of the complication. Descriptive data are presented as numbers and percentages for categorical variables. Continuous variables are presented as medians and interquartile ranges or means and standard deviations. REDCap^®^ was used for data collection and IBM SPSS Statistics version 28.0.1.0 was used for data analysis.

## Results

### Patient demographics

The median age of patients at HR was 59 (range 29–83) years. Nineteen patients (68%) were men, and nine patients (32%) were women.

### Rib fractures and rib fractures fixed

In our study, we identified a total of 346 rib fractures. The median number of fractured ribs per patient was 12, ranging from 1 to 39 rib fractures. We fixed a total of 153 rib fractures, and the median number of ribs fixed per patient was 5 (range 1–21). 91% clawing clips and 9% titanium plates with screws were used. 20 bridging clip systems were used in 6 patients. This system can treat multiple rib fractures. The number of rib fractures and ribs fixed per patient within each indication group are described in Table 1.

**Table 1:** Rib fractures and rib fractures fixed per patient within each indication group

	All patients *n* = 28	Thoracic pain *n* = 10	Discomfort *n* = 7	Chest tightness *n* = 6	Hardware dislocation *n* = 3	Hardware infection *n* = 2
Rib fractures; n, median	11.5	10.5	12	12	12	12.5
Rib fractures fixed	5	5	9	4.5	5	3

n, number

### Surgery for hardware removal

The overall median time from implantation to HR was 18 months (range 5 days-104 months). The intervals between the SSRF and HR for each indication group are described in Table 2. We removed a total of 96.5 implants, resulting in a median of 3 implants per patient (range 0.5-8). Of the 28 patients included, complete HR was performed in 22 patients (79%), and incomplete HR was performed in 6 patients (21%). The reasons for incomplete HR were localized thoracic pain (2), localized discomfort (2), localized unilateral discomfort in one patient who underwent bilateral rib fixation (1) and inadequate plate length (1). In the group of patients with complete HR, one patient with hardware infection underwent a two-stage hardware removal procedure during hospitalization.

**Table 2:** Time interval from surgical stabilization of the rib fracture to HR surgery

	All patients *n* = 28	Thoracic pain *n* = 10	Discomfort *n* = 7	Chest tightness *n* = 6	Hardware dislocation *n* = 3	Hardware infection *n* = 2
Time interval; month, median	18.2	18.5	18.2	22.3	8.6	0.9

n, number

### In-hospital complications related to hardware removal surgery

We observed 4 in-hospital complications related to HR surgery in 4 patients (14%). One new rib fracture was identified on X-ray imaging on the day of HR surgery and was treated conservatively. In one patient, a secondary rib fracture dislocation occurred during HR surgery performed five days after SSRF due to hardware-related infection. After this fracture was refixed with monofilament absorbable sutures, we did not observe rib fracture dislocation on further imaging. Pneumothorax was observed in one patient after HR and required chest tube placement. Finally, a peripheral bronchopleural fistula caused by surgical lysis of the adhesions between the visceral and parietal pleura during surgery occurred in one patient with an implant-associated infection. This infection with rib osteomyelitis caused adhesions between the two pleura sheets. After removal of this plate and partial rib resection, a small pleural defect developed. The bronchopleural fistula was closed with a monofilament absorbable suture. The in-hospital mortality rate was 0%. All complications were graded according to the Clavien-Dindo classification [24] and are described in Table 3.

**Table 3:** Complications that occurred during HR surgery according to the Clavien-Dindo classification

	All patients *n* = 28	Clavien-Dindo classification
New rib fracture during surgery; n	1	I d
Peripheral bronchopleural fistula	1	III a
Pneumothorax	1	III a
Secondary rib fracture dislocation	1	III b

n, number

### Indications for HR

#### Thoracic pain

Out of a total of 10 patients in this group, 6 patients attended the study-specific follow-up visit. Two patients described persistent thoracic pain as diffuse, and four patients described it as localised thoracic pain. The two patients with diffuse pain experienced bilateral thoracic trauma with complex rib fractures and received extensive SSRF. Both patients underwent complete HR. These patients reported pain relief; however, persistent thoracic pain was still present. Two patients with localized pain underwent delayed SSRF 3 and 8 weeks after the initial trauma. In both cases, rib fracture union was not observed on CT before SSRF. They reported persistent neurogenic pain after SSRF and were treated in a pain clinic. We performed neurolysis during HR surgery in both. However, these patients reported persistent pain after HR. The other two patients who underwent SSRF in two locations, lateral and dorsal, reported localized pain over the fixation material without neurogenic pain and underwent a partial HR in only one location. These patients reported significant pain relief after HR.

#### Discomfort

Defined as an unpleasant feeling without thoracic pain or chest tightness in patients with an intact fixation device. Among a total of 7 patients, 6 attended the study-specificfollow-up visit. Three patients experienced localized discomfort, and in two patients, we performed incomplete hardware removal. One of these patients, who had undergone bilateral rib fracture fixation for severe polytrauma following a bull attack, including bilateral flail chest, resulting in 39 rib fractures in multiple anatomical sectors, developed unilateral discomfort caused by extensive subscapular neobursa formation. The third patient had only one rib implant, which was removed. Three other patients reported diffuse discomfort, and we removed all the rib implants. In one of these patients, we observed palpable rib fixation material with a reduced subcutaneous fat layer who requested hardware removal.

#### Chest tightness

The presence of a subjective feeling of chest stiffness or restricted chest wall expansion was defined as chest tightness. Four patients with chest tightness were admitted with polytrauma, three after a traffic accident and one after a fall from a height. Two other patients experienced monotrauma, one after a traffic accident and the other after a fall from the stairs. All patients in this indication group underwent SSRF with clawing clips, and none of them underwent rib fixation with plates. After hardware removal, two-thirds of the patients experienced improvement, whereas one-third experienced a persistent corset feeling. We did not observe a correlation between the incidence of chest tightness and the severity of thoracic trauma; the number of rib fractures and rib fixations in this indication group was not greater than that in the other groups. Furthermore, none of the patients with chest tightness developed extensive callus formation, which could have contributed to the development of chest tightness. The causal factors that could explain the development of this phenomenon in our small series cannot be assessed.

#### Hardware dislocation

We observed 3 patients with hardware dislocation. One patient developed dislocation of one titanium plate due to inadequate plate length. In this patient, we performed partial removal of one plate including three screws because the other parts were not dislocated, and the rib fracture was consolidated at the time of HR surgery. In the other two patients, we observed partial clip dislocation caused by insufficient fixation of the claws to the ribs. This outstanding clip, located under the scapula, caused the formation of a subscapular neobursa, which in both cases subjectively manifested as unpleasant rubbing sensations in this region.

#### Hardware infection

This condition occurred in two patients. One patient experienced blunt chest trauma after a fall from a height while abroad and subsequently underwent repeated surgery with chest tube insertion and atypical lung resection. After repatriation to our hospital three weeks after the trauma, he was diagnosed with empyema. We performed evacuation of the empyema and SSRF of two rib fractures with nickel-titanium clips because of pain and dislocation 23 days after the trauma. However, we had to remove both clips 5 days after SSRF because of persistent infection and reduced the degree of dislocation with monofilament absorbable sutures. We did not obeserve any signs of an allergic reaction. Repeated vacuum-assisted closure (VAC) therapy was needed, but the patient was discharged 53 days after hospital admission, and we did not observe any nonunion development. The other patient suffered polytrauma during a high-velocity accident. After treating life-threatening injuries, we performed an SSRF with 4 titanium plates 3 days after the trauma. Ten days later, this patient developed parapneumonic empyema leading to respiratory insufficiency with progression to acute respiratory distress syndrome requiring endotracheal intubation and mechanical ventilation. As a consequence of pneumonia, the patient developed a hardware-related infection and required repeated surgery with VAC therapy and finally removal of titanium plates. On day 15 after SSRF, we performed wound revision and VAC therapy without HR to provide the chance for the rib fracture to heal. We removed one plate on day 50 during reoperation because of hardware infection due to a completely unstable rib fracture under the plate. Owing to the development of rib osteomyelitis, we performed partial rib resection, which resulted in an unavoidable chest wall defect. All three remaining plates were removed on day 77 due to osteosynthesis-associated infection after multiple reoperations. After a complicated course with repeated VAC exchanges and latissimus flap chest wall reconstruction, the patient was finally discharged 103 days after admission. All indications for HR are described in Table 4.

**Table 4:** Indications for HR surgery after surgical stabilization of rib fractures

	Patients included in the study *n* = 28	Patients with health status survey follow up *n* = 23
Thoracic pain; n (%)	10 (36)	6 (26)
Discomfort	7 (25)	6 (26)
Chest tightness	6 (21)	6 (26)
Hardware dislocation	3 (11)	3 (13)
Hardware infection	2 (7)	2 (9)

HR, hardware removal; n, number; %, percent

### Long-term outcomes of health status and patient satisfaction

The study-specific follow-up visit was performed 28 months (range 3–72) after HR. Out of a total of 28 eligible patients, 23 patients (82%) attended the follow-up visit to complete the study-specific health survey, and to answer additional questions.

#### Health status survey part I

**Fig. 1:**
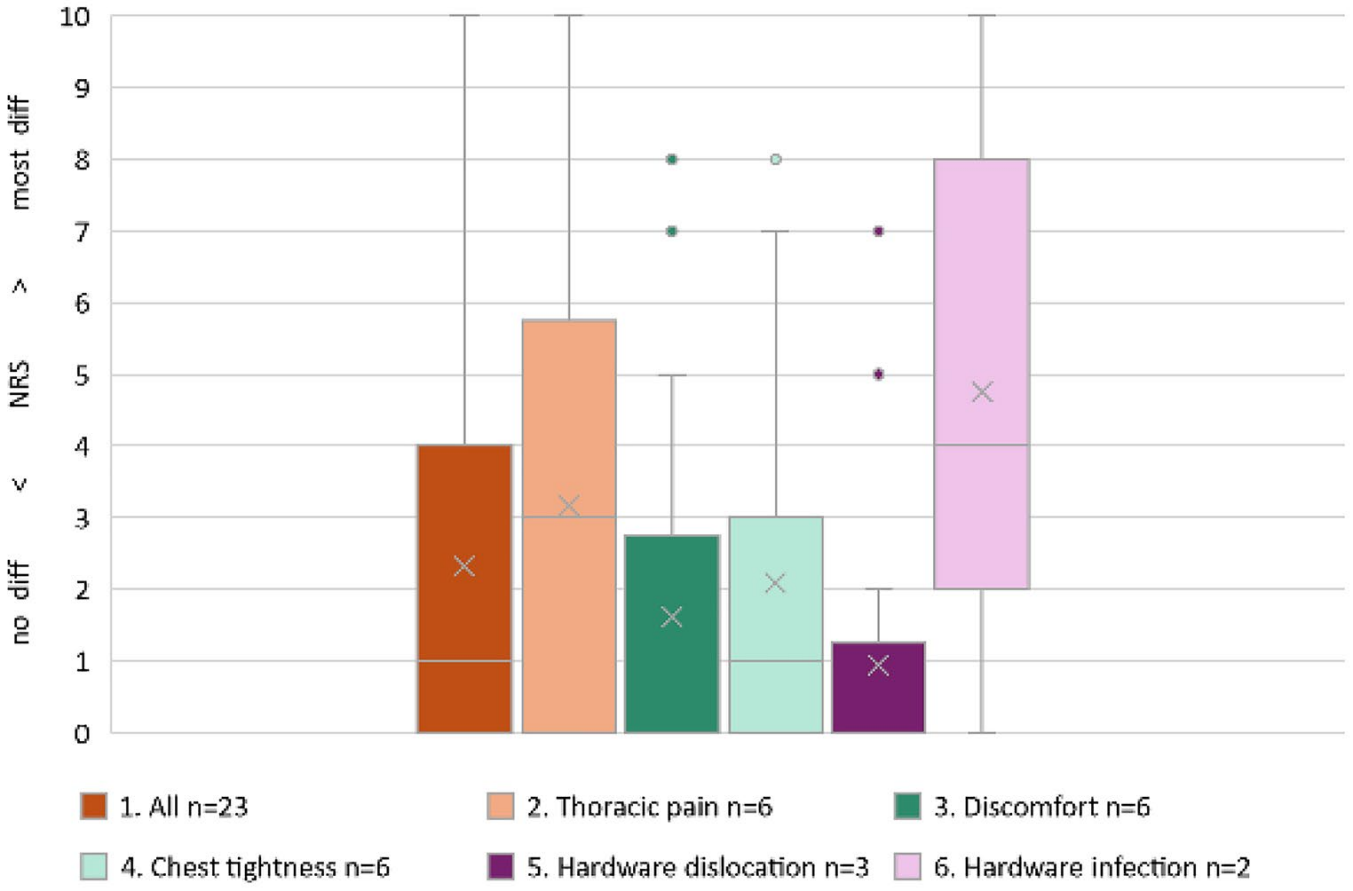
Health Status Survey Part I. Box plot distribution of global health status for all follow-up participants and in each indication group. The bottom and top of the boxes represent the first and third quartiles, the horizontal bar within the boxes represents the median and the cross determines the mean. An increasing NRS score corresponds to increasing difficulty: a value of 0 indicates “no difficulty present”, and a value of 10 indicates “most difficulty present”

**Fig. 2:**
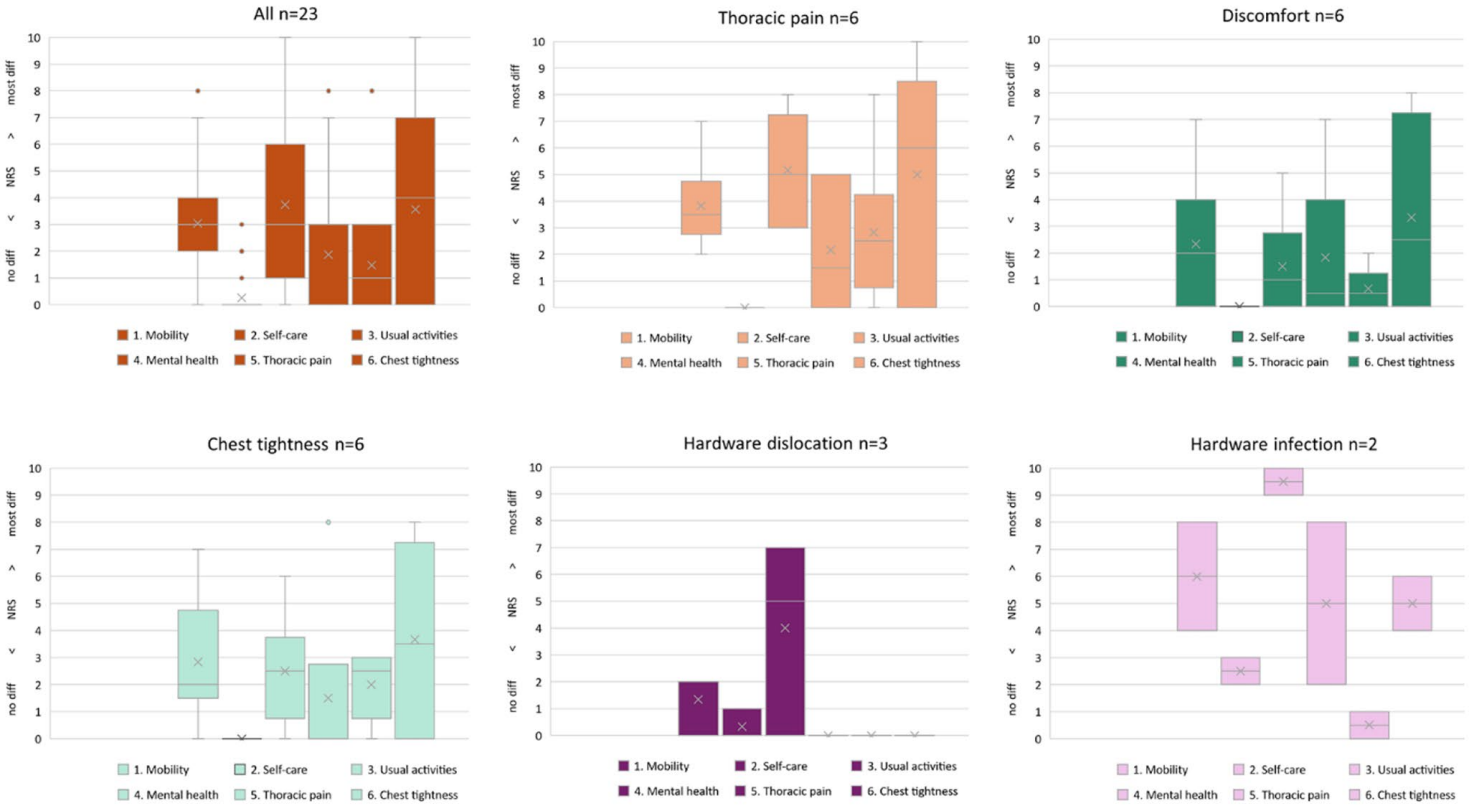
Health Status Survey Part I. Box plot for health status in each functional dimension scale (mobility, self-care, usual activities, and mental health) and symptom dimension scale (thoracic pain, chest tightness) of the study-specific health survey in all follow-up participants and in each indication group

The long-term health status outcomes from the study-specific health survey functional dimension scales and the symptom dimension scales are reported as global health. In the first part of our survey, the best median global health score (NRS 0) was observed in the discomfort and hardware dislocation indication groups, and we observed no difficulties. Patients with thoracic pain and hardware infection reported moderate health difficulties in which the median NRS scores were 3 out of 10 and 4 out of 10, respectively. In the chest tightness group, the NRS score was 1, which was equal to the average score of all 23 patients who attended the follow-up visit. Previously employed patients resumed their professional activities with mild to moderate problems (median NRS 4/10). Overall patient satisfaction with the cosmetic results was high (NRS 2/10). Furthermore, patients were very satisfied with the decision for surgery and would undergo HR surgery again (NRS 0/10). All the results are described in Table 5 and shown in Figs. 1 and 2.

**Table 5:** Descriptive analysis of health status survey part I: Patient-reported long-term outcomes at the study-specific follow-up visit in terms of health status using a study-specific health survey

	All patients *n* = 23	Thoracic pain *n* = 6	Discomfort *n* = 6	Chest tightness *n* = 6	Hardware dislocation *n* = 3	Hardware infection *n* = 2
Functional dimension scales [NRS 0–10]						
Mobility	3	3.5	2	2	2	6
Self-care	0	0	0	0	0	2.5
Usual activities	3	5	1	2.5	5	9.5
Mental health	0	1.5	0.5	0	0	5
Symptom dimension scales						
Thoracic pain	1	2.5	0.5	2.5	0	0.5
Chest tightness	4	6	2.5	3.5	0	5
Global health status	1	3	0	1	0	4
Additional dimension scales						
Resumption of professional activities	3.5	4	1	3	5	10
Satisfaction with the cosmetic result	2	4.5	2.5	1.5	0	3
Satisfaction with the decision for surgery	0	0.5	0	0	0	0

n, number; NRS, numerical rating scale; scale ranging from 0 to 10; higher NRS score indicates more difficulties; median values indicated; global health status is the median sum of the functional and symptom dimension scales in terms of health status

#### Health status survey part II

Patients with chest tightness and hardware infection showed the greatest improvement in global health status (75%) on the functional and symptom dimension scales, followed by the hardware dislocation group (61%). In the two other indications groups, the improvement rate was 58%. The improvement for all patients (*n* = 23) was 65%. Patients with hardware dislocation were the indication group with the highest improvement ratio (50% much better). In comparison, most patients with chest tightness experienced only a slight improvement in global health (42%) since the hardware was removed. The most frequent indication group reported no change in health status was in the discomfort group (42%). Patients with persisting thoracic pain were the only indication group that showed a 17% worsening of health status at the post-HR follow-up visit compared with that before HR.

**Fig. 3:**
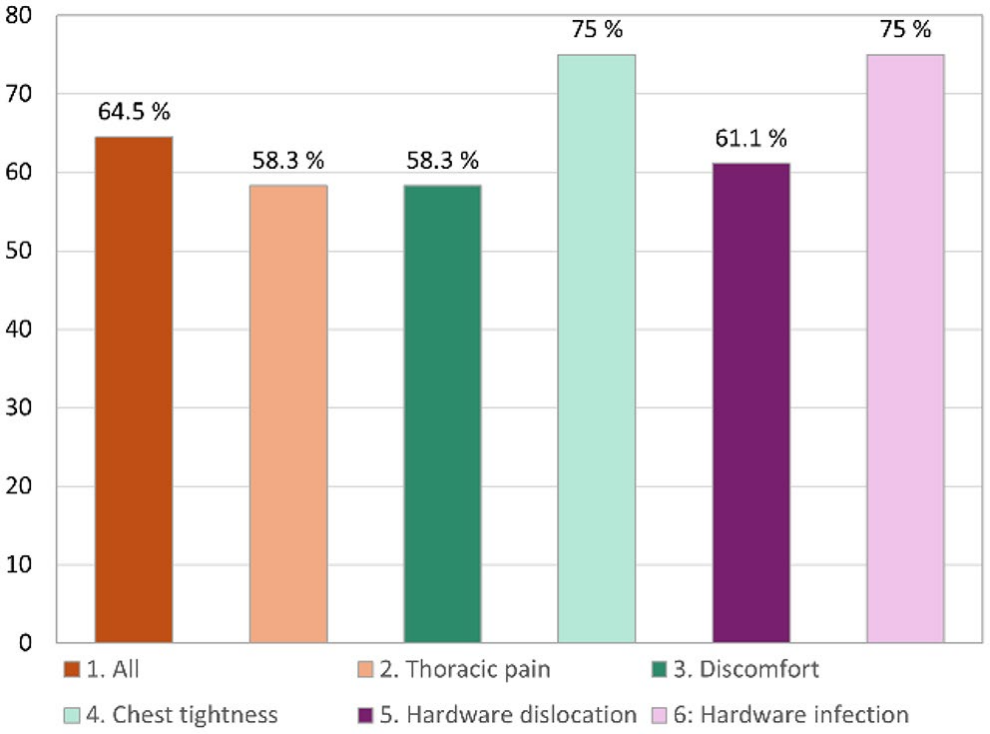
Health Status Survey Part II. Bar plot distribution of global health status improvement as a percentage for all HR follow-up participants and in each indication group compared with the health status before HR

**Fig. 4:**
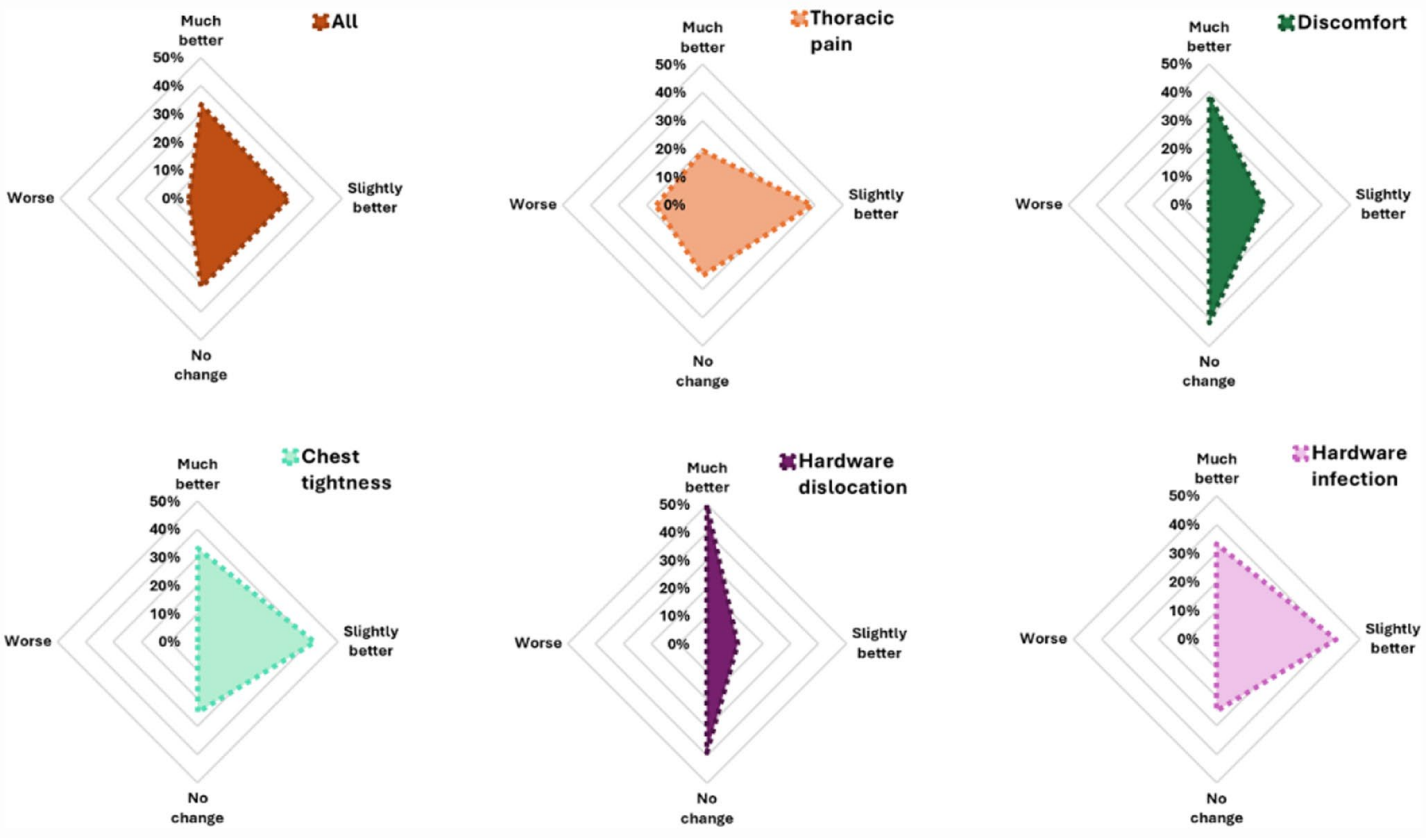
Health Status Survey Part II. Radar charts for change in health status in four categories expressed in percentages: much better, slightly better, no change, worse in the study-specific health survey in all HR follow-up participants and in each indication group

In summary, nearly two-thirds (65%) of all patients benefited from HR with improvement in their symptoms (much better rather than slightly better). All results are described in Table 6 and shown in Figs. 3 and 4.

**Table 6:** Descriptive analysis of the health status survey part II: Patient-reported long-term outcomes at the study-specific follow-up visit in terms of change in health status after HR compared with the status before HR

	All patients *n* = 23	Thoracic pain *n* = 6	Discomfort *n* = 6	Chest tightness *n* = 6	Hardware dislocation *n* = 3	Hardware infection *n* = 2
Functional dimension scales [%]						
*Mobility*						
Much better	34.8	16.7	50	16.7	66.7	50
Slightly better	39.1	66.7	16.7	66.7	0	0
No change	21.7	0	33.3	16.7	33.3	50
Worse	4.3	16.7	0	0	0	0
*Self-care*						
Much better	39.1	16.7	50	33.3	66.7	50
Slightly better	26.1	50	16.7	33.3	0	0
No change	30.4	16.7	33.3	33.3	33.3	50
Worse	4.3	16.7	0	0	0	0
*Usual activities*						
Much better	43.5	33.3	50	50	66.7	0
Slightly better	21.7	33.3	16.7	16.7	0	50
No change	30.4	16.7	33.3	33.3	33.3	50
Worse	4.3	16.7	0	0	0	0
*Mental health*						
Much better	21.7	16.7	16.7	16.7	33.3	50
Slightly better	30.4	16.7	33.3	50	0	50
No change	43.5	50	50	33.3	66.7	0
Worse	4.3	16.7	0	0	0	0
Symptom dimension scales						
*Thoracic pain*						
Much better	34.8	16.7	50	33.3	66.7	0
Slightly better	47.8	50	16.7	66.7	33.3	100
No change	13	16.7	33.3	0	0	0
Worse	4.3	16.7	0	0	0	0
*Chest tightness*						
Much better	26.1	16.7	16.7	50	0	50
Slightly better	21.7	16.7	16.7	16.7	33.3	50
No change	47.8	50	66.7	33.3	66.7	0
Worse	4.3	16.7	0	0	0	0
Global health status						
Much better	33.3	19.4	38.9	33.3	50	33.3
Slightly better	31.2	38.9	19.4	41.7	11.1	41.7
No change	31.2	25	41.7	25	38.9	25
Worse	4.3	16.7	0	0	0	0

n, number; %, percent; global health status is the sum in percent of the functional and symptom dimension scales in terms of change in health status

## Discussion

To our knowledge, this is the first systematic evaluation of long-term outcomes in patients who underwent HR after SSRF using a study-specific health survey.

### Locations of HR

According to current studies, the prevalence of HR after SSRF for any reason is between 1.7% [16] and 9% [12]. Our study revealed a comparable trend. The most frequent site of HR in our series was the lateral sector (see Appendix Figs. 5, 6 and 7), which corresponds to the most frequent location of rib fractures and implantation of rib stabilization hardware. These findings are in accordance with the results of a study published by Liebsch et al. [25] identifying the patterns of rib fractures after blunt chest trauma. Overall, the hotspots for the occurrence of rib fractures were at rib levels 4 to 7 of the lateral segments. In addition, several studies using titanium plates reported hardware failures in the lateral and posterolateral sectors [16, 26, 27].

**Fig. 5:**
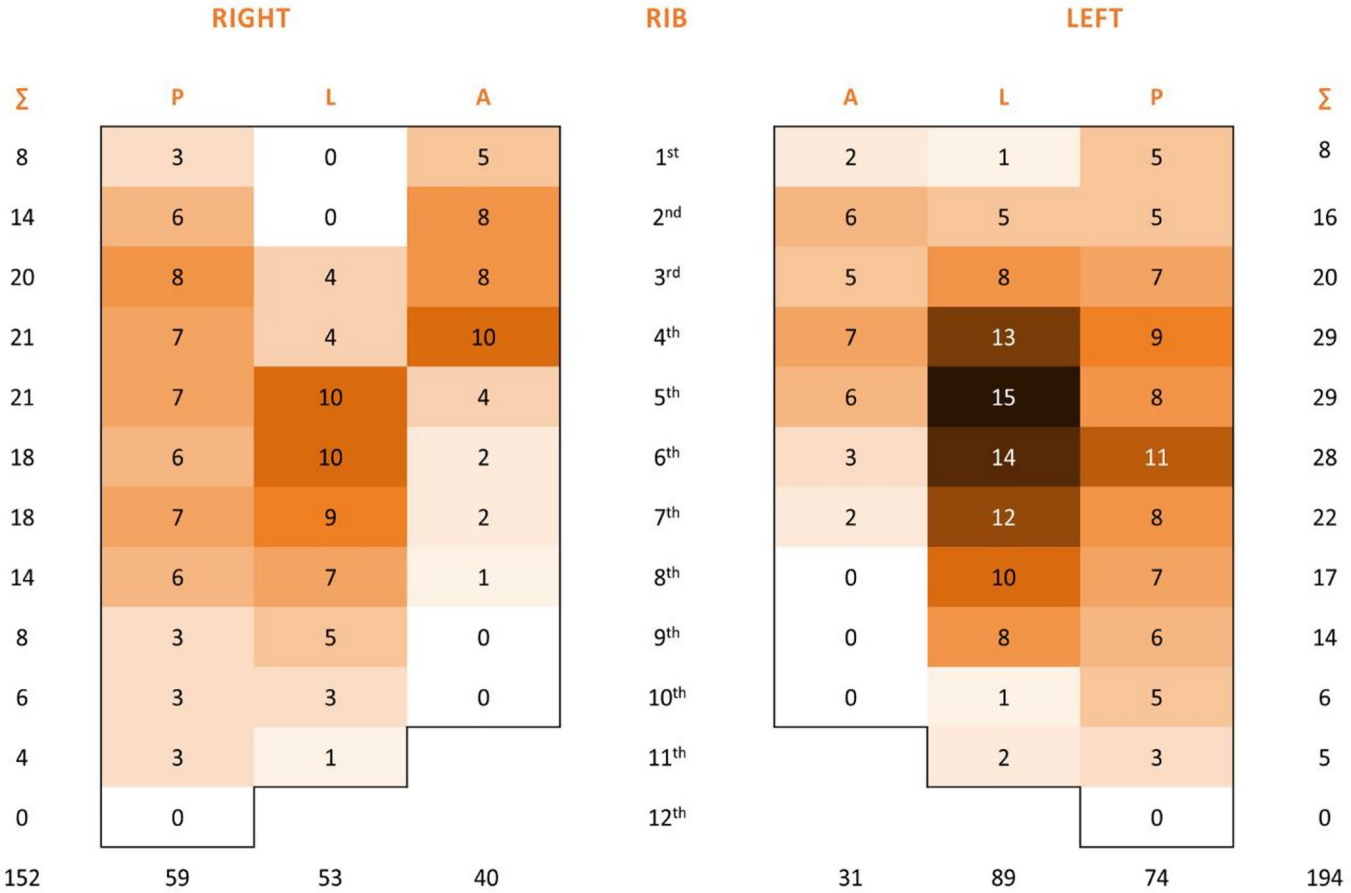
Heatmap demonstrating the distribution of rib fractures in 3 anatomical sectors: posterior, lateral, and anterior for each rib and site

**Fig. 6:**
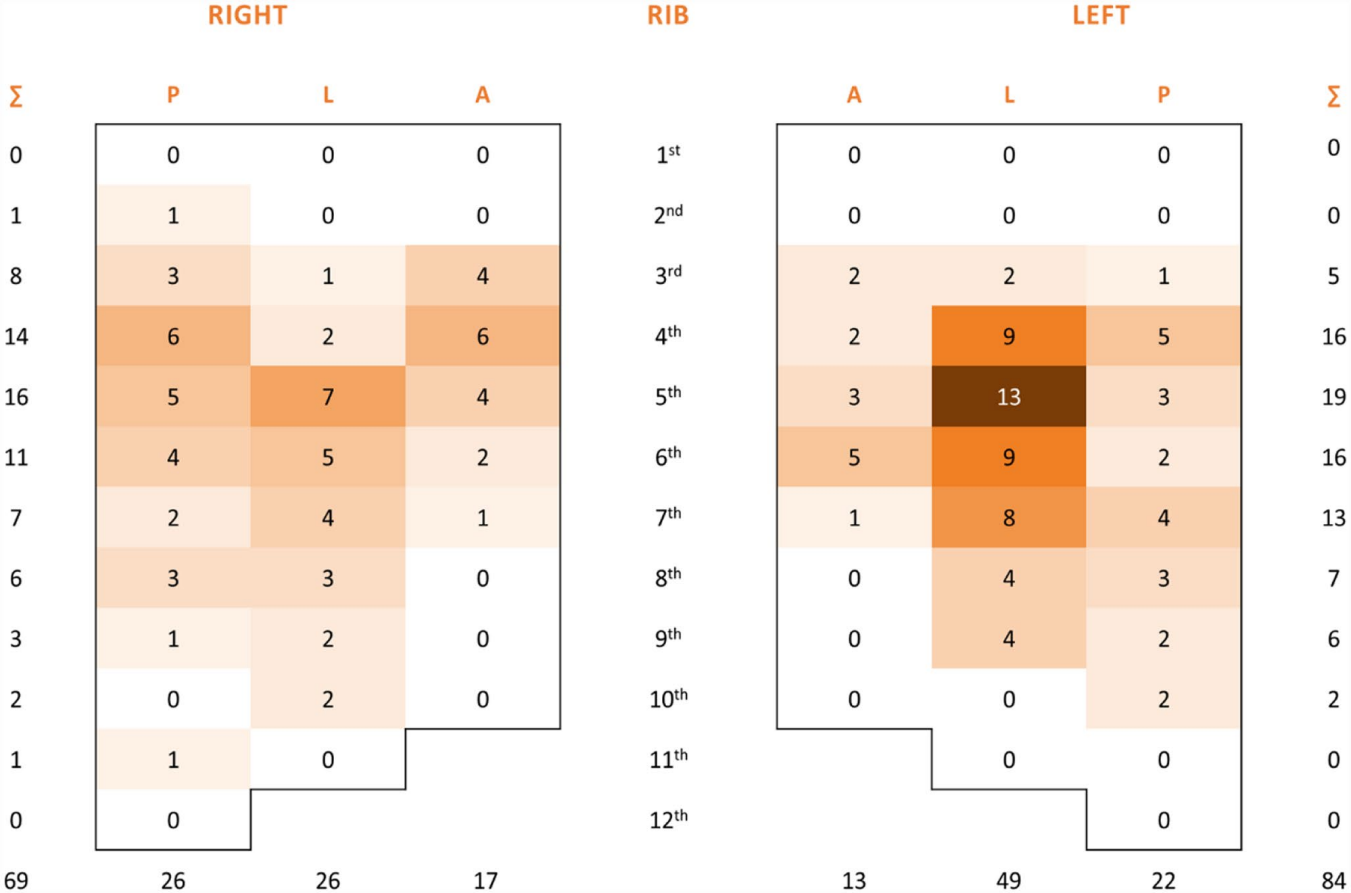
Heatmap demonstrating the fixed rib fracture distribution in 3 anatomical sectors: posterior, lateral, and anterior for each rib and site

**Fig. 7:**
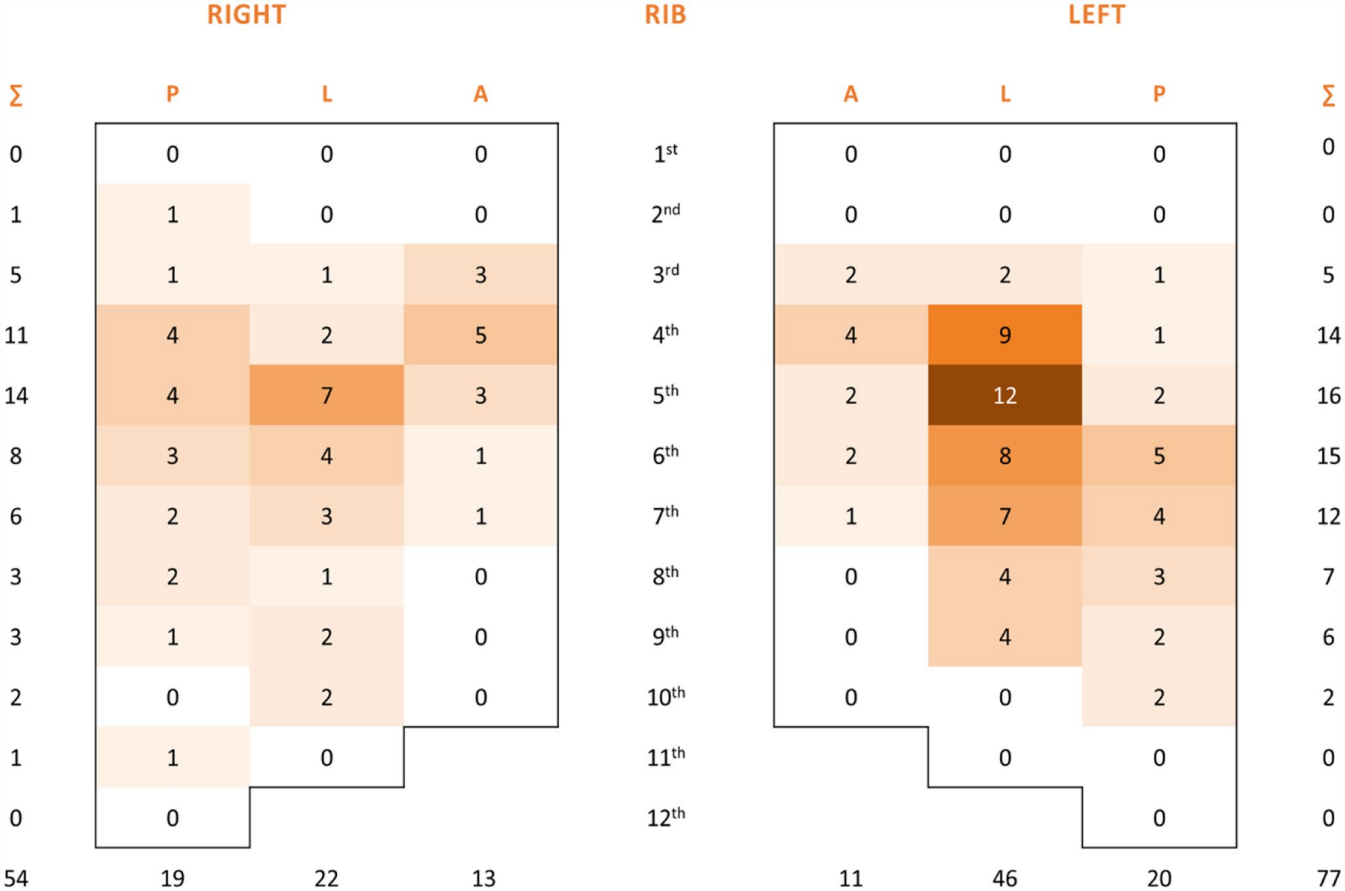
Heatmap demonstrating the rib fracture distribution of removed hardware in 3 anatomical sectors: posterior, lateral, and anterior for each rib and site

### Indications for HR

The main indications for HR after SSRF in our study were thoracic pain, discomfort, and chest tightness. We used 91% clawing clips and 9% titanium screwing plates. These findings are consistent with other studies reporting that ongoing thoracic pain, discomfort, and implant irritation [12, 14, 18, 28, 29] were the main causes of removal of rib stabilization hardware. Other studies using only titanium plates with locking screws reported mechanical failure, such as broken/displaced plates [16, 30, 31], dislocated rib splints [32] or lost screws [19, 33], as the main cause of HR after SSRF. Regarding the influence of the material used on the incidence of complications leading to HR in our 28 patients, no statement could be made, as no statistical analysis can show significance in the five indication groups.

### Thoracic pain

Trauma is reported as the primary cause of chronic pain in 12–18% of patients [34]. Shelat et al. [35] reported that 22.5% of patients experienced persistent thoracic pain at least 1 year after traumatic rib fractures. In addition, 26% of patients required regular use of analgesics, 35% complained of impaired professional activity, and 13% complained of impaired personal quality of life. The prevalence of chronic pain was not related to age, number of rib fractures, flail chest, injury severity score, occurrence of hemothorax or pneumothorax, or chest tube insertion. These findings are in accordance with our study. There was no relationship with the number of rib fractures, flail chest or trauma severity in general. The worst outcomes were observed in patients with a clear absence of rib fracture union who underwent delayed SSRF and subsequent neurolysis during HR surgery. Similarly, Van Wijck et al. reported more pain intensity in patients after surgical stabilization of rib fracture nonunion where intercostal nerve intervention was necessary [36]. In our study, patients indicated for HR because of persistent thoracic pain had the worst health status outcomes and the worst improvement rate among all indication groups. It was unclear whether the persistent thoracic pain was caused by trauma or hardware; therefore, we postponed HR and performed it to relieve the distress of these patients. Today, we know that most patients with persistent thoracic pain and chest tightness could benefit from HR and that this procedure should be performed earlier. However, there is still potential for improvement, and the challenge is to identify which patients could benefit.

### Discomfort

Patients with discomfort often present with poor symptoms, which could lead surgeons to uncertainty in the decision to remove hardware and the preference for observation management [22]. Symptoms without the pressure of suffering inevitably led to a longer interval between SSRF and HR. This condition could explain why the improvement was lower than that in the other groups. Discomfort, together with hardware dislocation, was associated with the formation of subscapular neobursa caused by irritation of the subscapular implanted rib fixation material.

### Chest tightness

To date, it has not been satisfactorily explained whether chest tightness is caused by thoracic trauma or is a consequence of rib stabilization surgery. Peek et al. suggested that chest tightness could be caused by the high stiffness of a rigid plate system on a consolidated rib, which may restrict chest wall movement [14]. A previous study [37] revealed that patients who underwent HR after SSRF with titanium plates due to chest tightness did not have restricted pulmonary function 6 months after surgery. This finding suggests an important contribution of the subjective component to the manifestation of this phenomenon. On the other hand, Prins et al. [38] reported that chest tightness occurred in patients with rib fractures who underwent SSRF as well as in those who were treated conservatively. Two randomized studies described a beneficial effect of rib fixation with clawing clips in reducing chest tightness [39, 40]. In our study, two-thirds of patients in the chest tightness group reported relief of this phenomenon after HR. However, one-third of patients reported no improvement. These results suggest that chest tightness may be more likely to be hardware related. Nevertheless, at the HR follow-up visit, two-thirds of the participants reported that this was due to trauma rather than hardware. However, we did not observe a correlation between the occurrence of chest tightness and the severity of chest trauma. The number of fractured and fixed ribs in these patients was not greater than that in the other groups. Therefore, the etiology of chest tightness remains unclear.

### Hardware dislocation

Dislocation or breakage of hardware can be objectively verified on X-ray or CT scans, which is a clear indication for HR compared with other potential indications, such as thoracic pain or discomfort, which can sometimes lead to controversy in decision making by surgeons [22]. In our study, we observed one patient with a dislocation due to an inadequately long plate and two patients with dislocation of the clawing clips. All of these patients experienced strong irritation and dissatisfaction. The interval between SSRF and HR surgery was by far the shortest in comparison to the other groups. Therefore, earlier removal of hardware and thus earlier relief of symptoms could explain the favorable results.

### Hardware infection

Hardware infection after SSRF is rare but is often associated with a high morbidity ratio. This was the case in both of our patients with hardware infection. The prevalence of hardware infection after SSRF ranges from 0.4% [41] to 4% [42]. Recent studies reported that 60–70% of these patients underwent complete HR [17, 42]. Systemic antibiotics, bone, and soft tissue debridement, and eventual hardware removal represent traditional treatment strategies. However, early HR sometimes results in the occurrence of rib nonunion [42, 43]. Therefore, we initially performed only partial HR to avoid bone healing complications in one patient. In addition, hardware infection might become chronic. A recent study reported that hardware infection following SSRF was symptomatic for up to 17 months [43]. Both of the patients in this group underwent repeated surgeries, and their length of stay, as well as their stay in the intensive care unit, was longer than that of other patients in our study. These patients had to cope with limited postoperative mobility and try to resume their activities in a longer period of time than the other patients. These were the reasons for the worst health status, usual activities, mobility, and mental health of the patients in our study. Among several potential risk factors, such as insufficient soft tissue or muscle hematoma [44], diabetes, smoking status, and chronic obstructive pulmonary disease [42], only Junker et al. [17] reported a comorbidity with a statistically significantly greater prevalence in patients with infection of SSRF hardware. These patients had a greater average body mass index than those who did not develop a hardware infection (*P* = 0.04). Similarly, in our study, both patients with infected hardware were overweight.

### Limitations

Some of our data were collected retrospectively and therefore have potential biases. In addition, the number of patients included was small, and the indication groups were not completely balanced. The study-specific follow-up visit was performed at different intervals after HR, which could influence the long-term results. Finally, our study was limited by the absence of five eligible patients who could not be successfully contacted. However, our results provide new insight into patient outcomes after HR.

## Conclusion

Our results suggest that the removal of hardware after surgical stabilization of rib fracture in patients with blunt chest trauma is a safe and feasible procedure, resulting in significant relief of symptoms and improvement in health status in approximately two-thirds of patients. Consequently, the removal of hardware could be beneficial for indicated patients and might be performed earlier and more liberally if symptoms are disabling.

## Data Availability

No datasets were generated or analysed during the current study.
